# Long-Term Follow-Up and Prognostic Factors for Advanced Thymic Carcinoma

**DOI:** 10.1097/MD.0000000000000324

**Published:** 2014-12-02

**Authors:** Jun-xin Wu, Hui-qin Chen, Ling-dong Shao, Su-fang Qiu, Qian-yu Ni, Bu-hong Zheng, Jie-zhong Wang, Jian-ji Pan, Jin-luan Li

**Affiliations:** From the Department of Radiation Oncology (JW, HC, LS, SQ, QN, BZ, JW, JP, JL), Teaching Hospital of Fujian Medical University, Fujian Provincial Key Laboratory of Translational Cancer Medicine, Fujian Provincial Cancer Hospital, Fuzhou, China.

## Abstract

The aim of this study was to evaluate the long-term survival outcomes in patients with advanced thymic carcinoma and identify prognostic factors influencing the survival.

We retrospectively analyzed 90 consecutive patients with pathologically confirmed advanced thymic carcinoma (Masaoka III and IV) in our institute, from December 2000 to 2012. Age, sex, clinical characteristics, laboratory findings, Masaoka and tumor node metastasis staging, pathologic grade, and treatment modalities were analyzed to identify prognostic factors associated with the progress-free survival (PFS) and the overall survival (OS) rates. Statistical analysis was conducted using SPSS, version 19.0 (SPSS, Inc, Chicago, IL).

A total of 73 (81.1%) male and 17 (18.9%) female patients participated in the study. The median follow-up time was 75 months (range, 20–158 months). The 5-year PFS and OS rates were 23.6% (95% confidence interval [CI], 14.6%–33.8%) and 35.7% (95% CI, 25.1%–46.4%), respectively. The multivariate Cox regression model analysis showed that factors improving the PFS were the normal lactate dehydrogenase (LDH) level (*P* < 0.001), Masaoka III stage (*P* = 0.028), and radiotherapy (RT) (*P* < 0.001). The LDH (*P* < 0.001), T stage (*P* < 0.001), and the pathologic grade (*P* = 0.047) were independently prognostic of OS.

Long-term follow-up of the advanced thymic carcinoma showed poor outcomes of PFS and OS. LDH, Masaoka stage, and RT affected the PFS, and LDH, T stage, and pathologic grade seemed to affect the OS. Establishing a better staging system for predicting outcomes would be warranted.

## INTRODUCTION

Thymic epithelial neoplasm (TEN) is a relatively rare cancer with an annual incidence of 0.15 per 100,000 person-years.^1^ In addition, thymic carcinoma accounts for <1%–4% of cases of TEN.^2^ Thymic carcinoma are quite different from thymoma in clinical behavior. It often presents as advanced disease, which results in poor survival outcome.^3^ Nowadays, thymic carcinoma staging is often done paralleling with the Masaoka–Koga clinical staging system and tumor node metastasis (TNM) staging.^4,5^

Owing to the rarity, most of the publications in the medical literature are small sample retrospective analyses from single institutions.^2,6^ Several studies suggested that tumors of low grade had a relatively favorable clinical outcome and a low incidence of local recurrence and metastasis.^6–8^ The ideal treatment modality for thymic carcinoma is unknown. The complete resection remains the cornerstone of successful treatment. Nevertheless, the diagnosis of advanced disease in the beginning precludes complete resection. The multidisciplinary therapy including resection, radiotherapy (RT), and chemotherapy (CT) would be recommended to the advanced thymic carcinoma for better control; however, the impact of these modalities on clinical outcome is unclear. RT seems to improve the local control, but the survival benefit remains uncertain.^3,6^ Ogawa et al^6^ reported that the patients received complete resection and adjuvant radiation of 50 Gy had no local recurrence,^6^ whereas Kondo and Monden^3^ reported no survival benefit from adjuvant RT within a retrospective study with small subgroup sizes. With the development of accurate radiotherapy (A-RT), RT might become more important in thymic carcinoma treatment. The CT is also based on a low-level evidence, with a few small studies of adjuvant cisplatin-based CT that achieved some beneficial effects.^9,10^ Overall, the survival prognosis and prognostic factors affecting survival have not been well investigated in advanced thymic carcinoma.

Here we retrospectively evaluated the clinical characteristics, long-term outcomes, and prognostic factors affecting survival in patients with advanced thymic carcinoma in our institution.

## METHODS AND MATERIALS

### Patients

We retrospectively analyzed a total of 90 consecutively treated patients with advanced thymic carcinoma in our institute, from December 2000 to 2012. This trial was approved by the ethics committee of Fujian Provincial Cancer Hospital, Fuzhou, China, in accordance with the Helsinki Declaration. All patients had given informed consents before treatment. All patients had pathologically confirmed thymic carcinoma, advanced disease (Masaoka III and IV), and no previous or concurrent malignancy. Pretreatment evaluation included a complete history, physical examination, complete laboratory tests, and staging.

We used the revised histologic classification proposed by Suster and Rosai who divided thymic carcinomas into high or low-grade lesions.^11^ The low-grade histology included well-differentiated squamous cell carcinoma, well-differentiated mucoepidermoid carcinoma, and basaloid carcinoma, and the high-grade histology included lymphoepithelioma-like carcinoma, small cell carcinoma, undifferentiated carcinoma, sarcomatoid carcinoma, and clear cell carcinoma.^11,12^ Staging of the cases was performed according to the Masaoka and TNM staging system, respectively.^4,5,13^ For patients who accepted surgical excision, the staging was determined by operative and pathological findings, and the person without surgery, the tumor, lymph node, and distant metastasis mainly relied on systemic physical examination, chest computed tomography scan, abdominal and pelvic ultrasound, brain magnetic resonance imaging and whole body bone emission computed tomography, and/or position emission tomography-computed tomography before any treatment.

### Statistical Analysis

The primary end point of this study was progress-free survival (PFS) and overall survival (OS). Age (≥60 vs <60 years), gender, pretreatment symptom (yes vs no), carcinoembryonic antigen (≥5 vs <5 ng/mL), lactate dehydrogenase (LDH) (≥190 vs <190 IU/L), alkaline phosphatase (ALP) (≥104 vs <104 IU/L), pretreatment anemia (yes vs no), pretreatment hypoproteinemia (yes vs no), ABO blood type (A type vs B/O type), pathologic grade (low vs high), T stage (T2 + 3 vs T4), N stage (N0 vs N1–3), M stage (M0 vs M1), Masaoka stage (IIIA + IIIB vs IVA + IVB), TNM stage (III vs IV), Masaoka IVB subgroup stage (the blood metastasis group, IVB-B vs the lymph node metastasis group, IVB-N vs non-IVB), surgery (yes vs no), surgical procedure (complete resection vs incomplete resection vs nonsurgery), RT (yes vs no), RT technique (conventional radiotherapy [C-RT] vs A-RT), RT dose (≥60 vs <60 Gy), CT (yes vs no), CT cycle (≥4 vs <4 cycles), and primary treatment method (postoperative chemoradiotherapy [post-CRT] vs chemoradiotherapy [CRT] vs CT alone) were analyzed to identify prognostic factors associated with PFS and OS, which were based on the response evaluation criteria in solid tumors 1.1 criteria.^14^ The PFS was defined as the interval from the date of primary treatment time to the date of disease progression, relapse, or death, and the OS was defined as the time between the date of diagnosis and death from any cause. Groups were compared using χ^2^ test. Survival curves were constructed using the Kaplan–Meier method and compared using log-rank tests. All tests of significance were 2 sided: differences at *P* values of <0.05 were considered to be significant. The Cox regression model was used to examine prognostic factors for PFS and OS. Statistical analyses were performed with SPSS statistical software package version 19.0 (SPSS, Inc, Chicago, IL).

## RESULTS

### Patient Characteristics

A total of 90 patients were included for the analysis in the present study. The patient's characteristics are shown in Table 1. The median age was 52 years (range, 11–80 years). There were 73 (81.1%) males and 17 (18.9%) females. There were 54.4% of the patients (49/90) who had a high pretreatment LDH level (≥190 IU/L) and 45.6% (41/90) who had normal level (<190 IU/L). Of all patients, 26 (28.9%) patients had stage III and the other 64 (71.1%) patients had stage IV disease. There were 45 (50%) patients identified as low-grade and 32 (35.6%) with high-grade, the other 13 (14.4%) patients could not be classified. Among the 48 patients who had ABO blood types test, 29.2% (14/48) were A type, 22.9% (11/48) were B type, 47.9% (23/48) were O type, and 0% (0/48) were AB type, respectively. Forty patients (44.4%) received surgery, in which only 9 (10.0%) patients received complete resection. There were 59 (65.6%) patients who received RT (31 C-RT, 28 A-RT). The rate of symptomatic radiation pneumonitis in C-RT and A-RT group was 12.9% (4/31) and 0.0% (0/28), respectively. There were 77 (85.6%) patients accepted for CT. The CT regimens were all based on platinum, with median cycles of 3 (range, 1–16 cycles). There were 64.4% (58/90) patients accepted for comprehensive treatment, including 35.6% (32/90) who received post-CRT, and 28.9% (26/90) who received CRT. Nineteen of the 90 patients received CT alone.

**TABLE 1:**
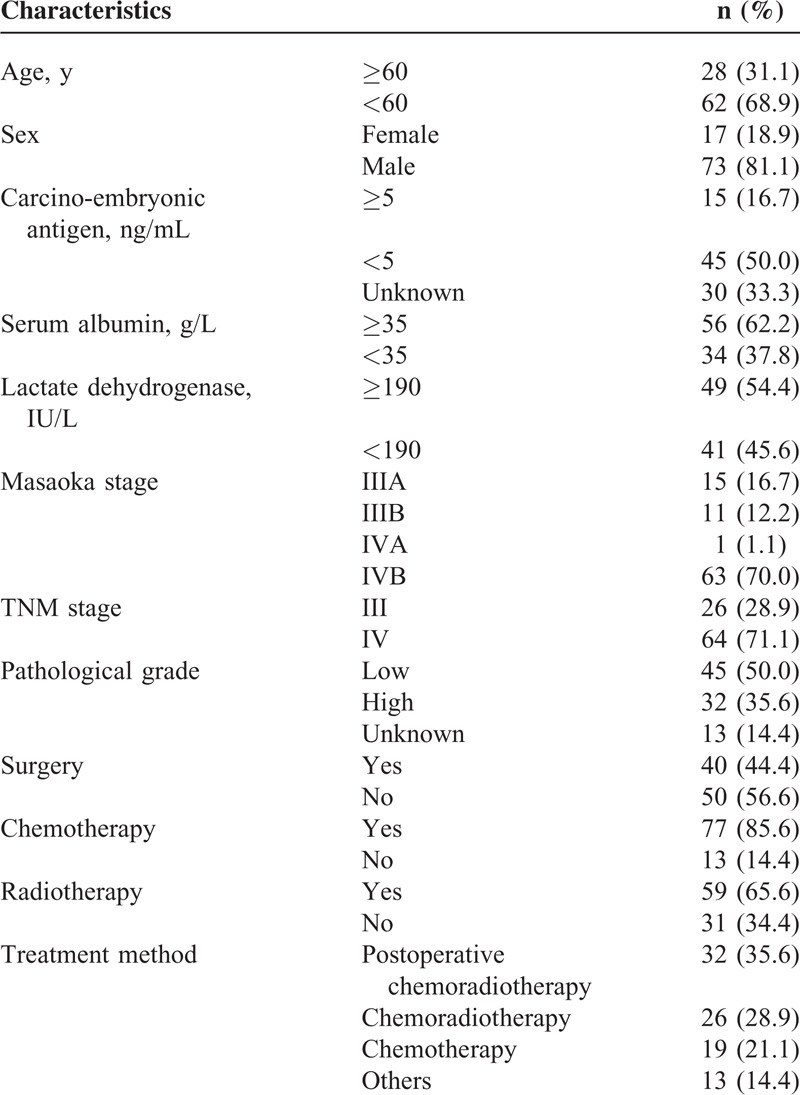
Patient Characteristics

### Survival Outcomes and Local Control

**FIGURE 1:**
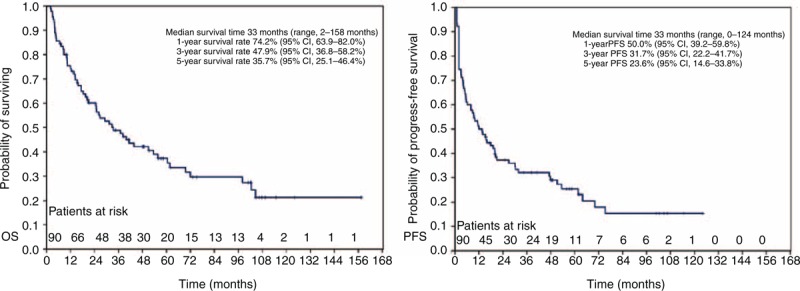
Kaplan–Meier analysis of (A) overall survival (OS) rates and (B) progress-free survival (PFS) rates in 90 patients with advanced thymic carcinoma. CI = confidence interval.

The median follow-up time was 75 months (range, 20–158 months). The median survival time was 33 months (range, 2–158 months). The median progression time was 12 months (range, 0–124 months). Sixty-nine (76.7%, 69/90) patients experienced progression, including 11 (12.2%, 11/90) with local recurrence, 41 (45.6%, 41/90) with distant metastases, and 17 (18.9%, 17/90) resistant to treatment without control. The locoregional recurrence included 4 (15.4%, 4/26) in CRT group and 6 (18.7%, 6/32) in post-CRT group. The common distant metastases sites included lung (18.9%, 17/90), liver (11.1%, 10/90), bone (11.1%, 10/90), and lymph node (14.4%, 13/90). The 1, 3, and 5-year OS for the whole group were 74.2% (95% CI, 63.9%–82.0%), 47.9% (95% CI, 36.8%–58.2%), and 35.7% (95% CI, 25.1%–46.4%), respectively (Figure 1A). The 1, 3, and 5-year PFS were 50.0% (95% CI, 39.2%–59.8%), 31.7% (95% CI, 22.2%–41.7%), and 23.6% (95% CI, 14.6%–33.8%), respectively (Figure 1B).

### Prognostic Factors Affecting Survival

**FIGURE 2:**
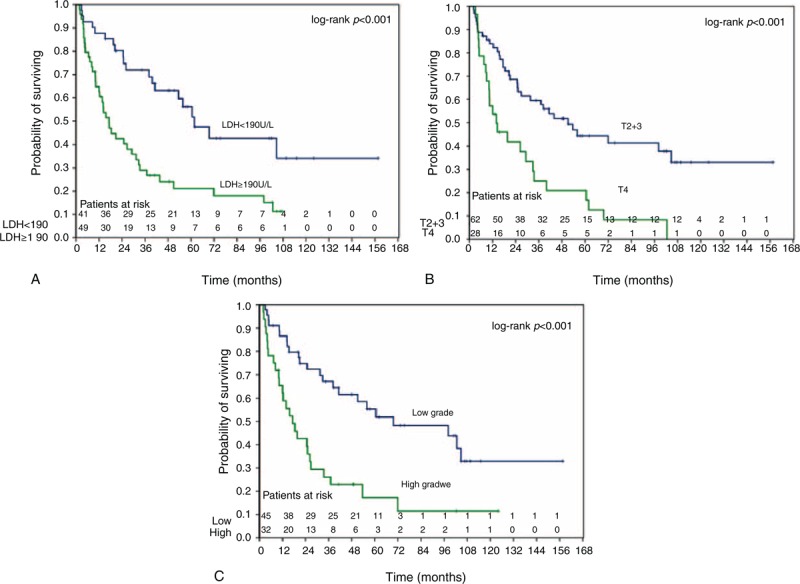
Kaplan–Meier analysis of overall survival rates in 90 patients with advanced thymic carcinoma according to (A) pretreatment LDH level (<190 IU/L: blue/≥190 IU/L: green); (B) T stage (T2 + 3: blue/T4: green); and (C) pathologic grade (low: blue/high: green). LDH = lactate dehydrogenase.

**FIGURE 3:**
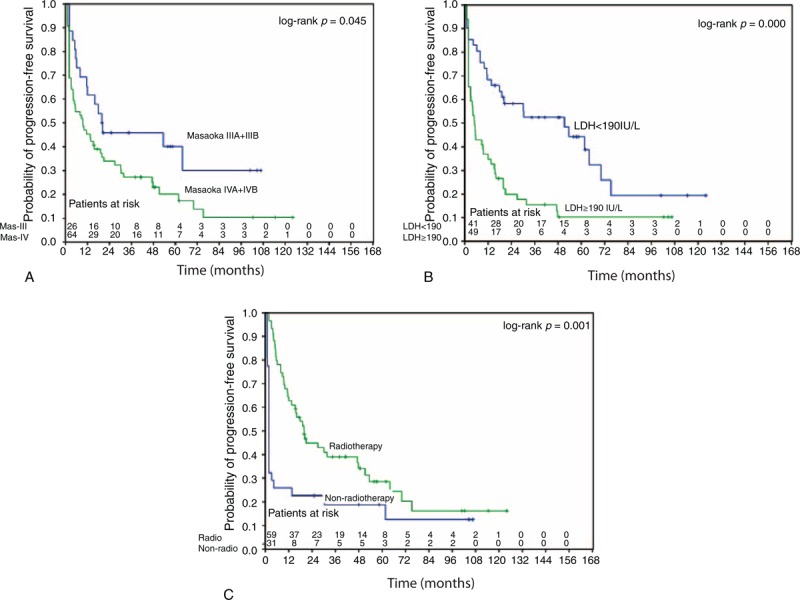
Kaplan–Meier analysis of progress-free survival rates in 90 advanced thymic carcinoma patients according to (A) Masaoka stage (IIIA + IIIB: blue/IVA + IVB: green); (B) pretreatment LDH level (<190 IU/L: blue/≥190 IU/L: green); and (C) radiotherapy (yes: green/no: blue). LDH = lactate dehydrogenase, RT = radiotherapy.

The univariate and multivariate analysis of factors influencing OS are summarized in Table 2. The univariate analysis showed that LDH, ALP, pathologic grade, T stage, M stage, Masaoka-IVB subgroup, surgery, and RT were significantly associated with OS (*P* < 0.05, respectively). The multivariate analysis showed that LDH (*P* = 0.004, hazard ratio [HR] = 2.787, 95% CI, 1.374%–5.652%), T stage (*P* = 0.001, HR = 3.134, 95% CI, 1.641%–5.985%), and pathologic grade (*P* = 0.047, HR = 1.964, 95% CI, 1.008%–3.827%) were independently prognostic of OS. Table 3 showed univariate and multivariate analysis of factors influencing PFS. The univariate analyses showed that LDH, T stage, M stage, Masaoka-IVB subgroup, Masaoka stage (III/IV), pathological grade, surgery, and RT were significantly associated with PFS (*P* < 0.05, respectively). Of these variables, LDH (*P* < 0.001, HR = 4.633, 95% CI, 2.319%–9.257%), Masaoka stage (*P* = 0.028, HR = 0.444, 95% CI, 0.216%–0.914%), and RT (*P* < 0.001, HR = 0.120, 95% CI, 0.050%–0.289%) were independently associated with PFS. Kaplan–Meier curves examining OS and PFS are presented in Figures 2 and 3.

**TABLE 2:**
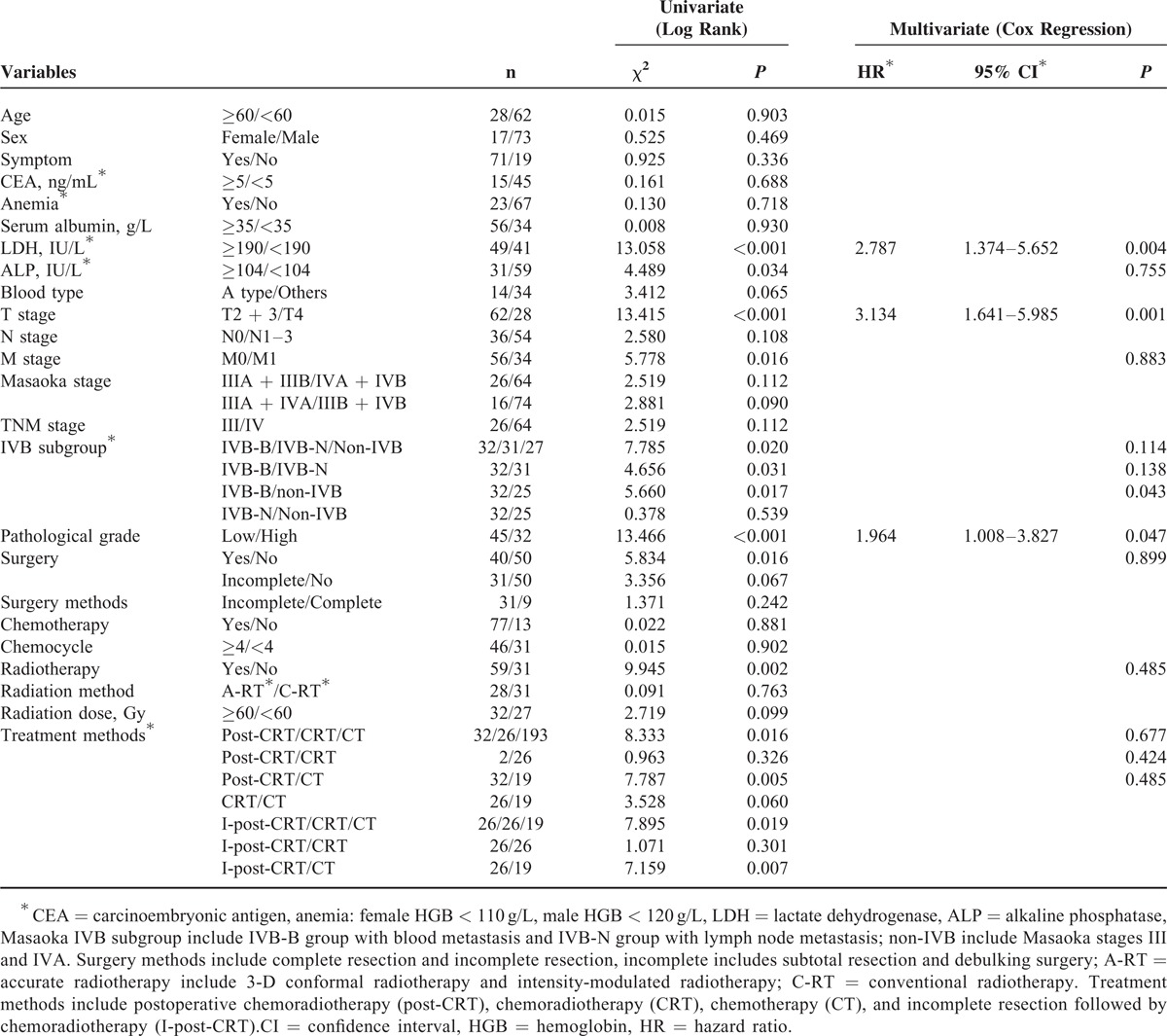
Univariate and Multivariate Analysis of Overall Survival

**TABLE 3:**
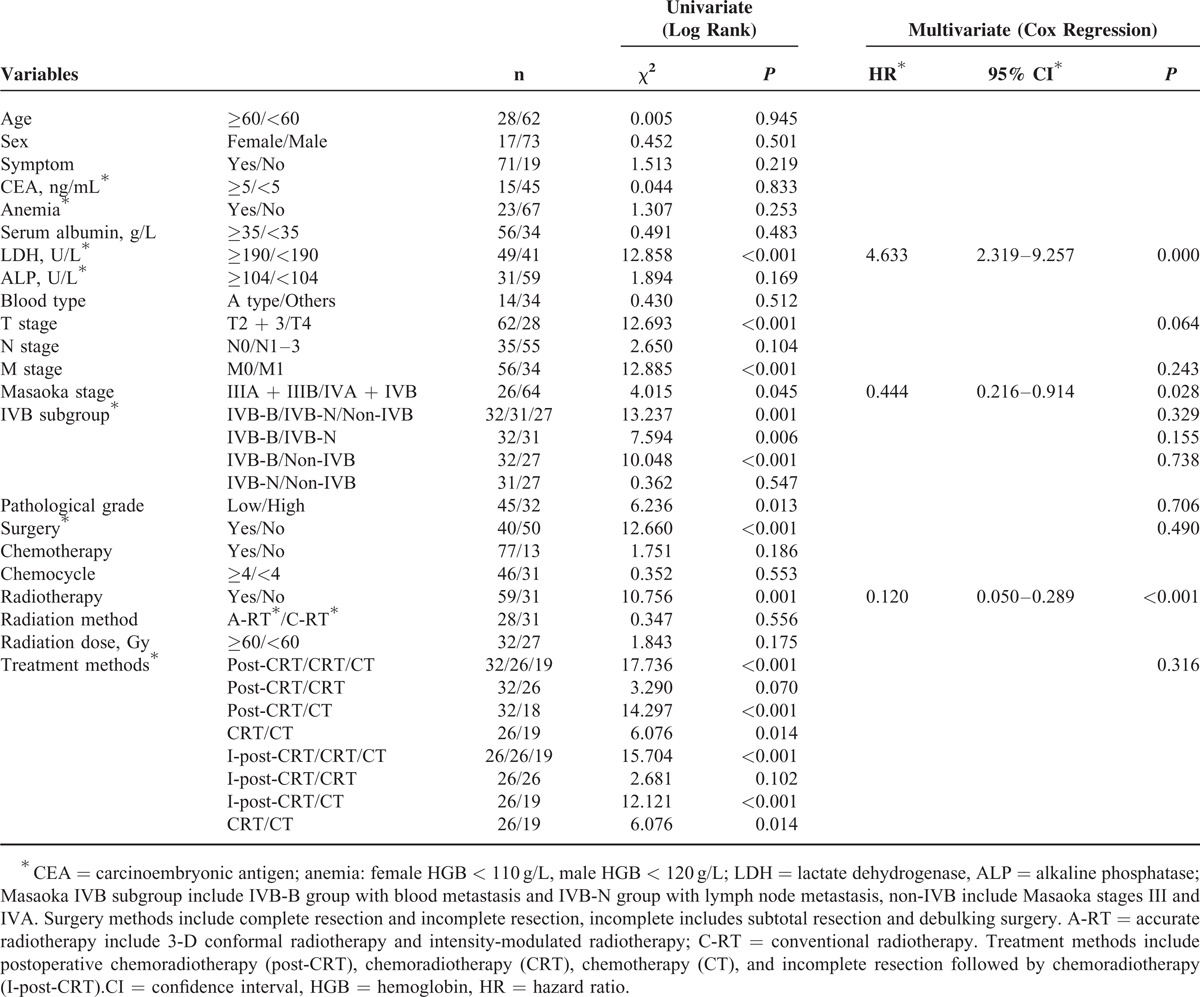
Univariateand Multivariate Analysis of Progress-Free Survival

## DISCUSSION

As one of the largest series of patients with advanced thymic carcinoma, the long-term follow-up revealed 5-year OS rate of 35.7% (95% CI, 25.1%–46.4%) and 5-year PFS rate of 23.6% (95% CI, 14.6%–33.8%). Only a few studies reported the 5-year PFS rate for thymic carcinoma because of its rarity.^15,16^ The 5-year OS of 30% was recently reported from another retrospective study.^17^ Our outcomes are quite similar to those previous studies. In addition, we also found that it seem to be a slight male preponderance (81.1%) that matched other series.^17,18^ The multivariate analysis showed that LDH, T stage, and pathologic grade were associated with OS, and LDH, Masaoka stage, and RT affected PFS. In addition, A blood type seems to have a better survival trend than B/O type.

The Masaoka stage has been reported to be a prognostic factor for the thymoma.^19^ However, whether the Masaoka stage could also be an independent prognostic factor for the thymic carcinoma is still controversial. The correlation between them has not been clearly clarified, especially for the advanced thymic carcinoma. Some authors agreed the prognostic value of the Masaoka stage,^17,20,21^ while other authors disagree.^6,12,22^ In addition, Tseng et al^22^ reported that prognosis of thymic carcinoma seemed to mainly rely on tumor invasion of the great vessels, but Masaoka stage. In our study of advanced thymic carcinoma, there is no statistical significant difference on 5-year OS between Masaoka stages III (43.0%, 95% CI, 22.1%–62.5%) and IV (32.9%, 95% CI, 20.9–45.4%) (*P* = 0.112), while further analysis showed that Masaoka stage might be the prognostic factor for PFS (stage III 33.9%, 95% CI, 14.2%–54.8% vs stage IV 17.3%, 95% CI, 8.3%–29.0%, *P* = 0.028).

Since previous reports had shown that there was <2% lymph node metastasis for the thymoma,^23^ the Masaoka–Koga staging system might not be fit to the highly metastatic thymic carcinoma.^24^ Yamakawa et al^5^ proposed a TNM system based on 207 patients,^5^ which was not widely used in thymoma because of the low lymph node metastasis,^25^ might be suitable to the aggressive thymic carcinoma. Some studies showed that the positive lymph node is a negative prognostic factor,^12,23,26^ while our study showed no difference on survival between N0 and N1–3 subgroup (*P* = 0.108). The advanced tumor staging and different diagnostic techniques might contribute to the different results. In our study, there was 61.1% (55/90) of patients diagnosed as positive lymph nodes, which was much higher than previous studies (16.2%–40%).^12,26^ Moreover, the Masaoka staging system focuses on the tumor itself, and classifies lymph node metastasis as stage IVB together with systemic metastasis.^27^ Here, we tried to divide Masaoka stage IVB into blood metastasis group (IVB-B) and lymph node metastasis group (IVB-N). We found that there was significant difference between IVB-N (39.8%, 95% CI, 20.1%–58.7%) and IVB-B (20.9%, 95% CI, 8.5%–37.0%) (*P* = 0.031) for the 5-year OS. In addition, there was no significant difference between non-IVB and IVB-N (*P* = 0.539). The IVB-N group seemed to have better survival than IVB-B group and similar survival with non-IVB group. According to the results above, lymph node metastasis might play a more important role in staging.

Similar to thymoma, it is confirmed by many studies that patients of thymic carcinoma receiving complete resection have better survival than those with either incomplete resection or without surgery.^3,17,18^ Kondo and Monden^3^ reported that the 5-year OS of total resection, subtotal resection, and inoperable groups were 67%, 30%, and 24%, respectively. As our study focused on the advanced thymic carcinoma, only 9 patients received complete resection. Of this group, 6 patients were still alive without any progression, and the other 3 patients died with progression (1 with local recurrence and 2 with distant metastases). The results would also suggest that the complete surgical resection might be one of the most important prognostic factors for advanced thymic carcinoma. On the contrary, whether advanced thymic carcinoma patients could benefit from incomplete resection (subtotal and debulking) remains controversial.^23,28^ The 5-year OS for the incomplete resection and nonsurgery in our study were similar to the studies above (39.1% vs 30% and 26.2% vs 24%). In addition, there was no 5-year OS benefit from incomplete resection in our study when comparing the incomplete post-CRT group (47.2%, 95% CI, 24.8%–66.8%) with CRT group (34.8%, 95% CI, 15.1%–55.4%) (*P* = 0.301).

Previous studies showed that the patients with advanced thymic carcinoma might benefit from RT.^29,30^ Ogawa et al^6^ reported the treatment results of 40 completely resected thymic carcinoma receiving a dose of 50 Gy as the postoperative RT, and found no local recurrence case. Another study reviewed 26 cases who underwent total or subtotal resection followed by postoperative RT alone, and found that the postoperative RT could achieve 5-year local control rate of 91%.^31^ Fan et al^32^ also analyzed 45 patients with advanced thymic carcinoma, and the study showed that the patients receiving RT appeared to have much better outcomes than those without. Our study showed that patients with advanced thymic carcinoma might have survival benefit from RT. The median survival time for patients in RT and non-RT groups were 51 months (range, 2–158 months) and 10 months (range, 2–108 months), respectively. The 5-year OS for the RT and non-RT groups were 42.5% (95% CI, 28.1%–56.2%) and 18.6% (95% CI, 7.0%–34.5%) (*P* = 0.002), respectively. In addition, the multivariate analysis showed that RT was the independent prognostic factor for PFS (*P* < 0.001). Moreover, there was no statistical difference in the survival rate between the C-RT and A-RT groups, while the rate of symptomatic radiation pneumonitis in the C-RT group (12.9%, 4/31) was higher than the A-RT group (0.0%, 0/28). Therefore, we would suggest A-RT to the advanced thymic carcinoma to minimize toxicities.

Most advanced patients (77, 85.6%) in our study received palliative CT. Unfortunately, thymic carcinoma responds poorly to CT, whether the CT can bring benefit for the thymic carcinoma and the gold CT regimens are still controversial because of the rare prospective trails.^2,23^ In our study, the CT did not show survival benefit. CT alone only achieved 5-year OS of 10% and PFS of 0%. However, the result may be limited by the small amount of data and the uneven sample proportion (only 13 patients did not accept the CT).

Similar with previous study, the pathologic grade was also found to be one of the most significant indicators of prognosis in this study.^23^ In addition, we also tried to find some biomarkers to predict prognosis. The Cox regression analysis of our study showed that the LDH level affect the advanced thymic carcinoma in both OS (*P* = 0.004) and PFS (*P* < 0.001). Therefore, we would suggest that the LDH level might also become a prognostic factor. Moreover, we also analyze the association between ABO blood types and outcomes of thymic carcinoma. The results showed that A type group seem to have a better survival outcome than B/O type group. The median survival time for A type group and B/O type group were 72 months (range, 2–158 months) and 25 months (range, 2–110 months), respectively. However, because of the limitation of the small sample, the survival and prognostic value of these findings must be further studied.

As a retrospective single-institution study, some limitations should be noted. Because of the rarity of this aggressive disease, the sample size remained small. In addition, only a few biomarkers were analyzed. Lacking details of immunohistochemistry would also prevent to find new genetic factors.

In summary, we have evaluated long-term survival outcomes and clinical prognostic factors affecting survival in patients with advanced thymic carcinoma. Although the survival outcomes remained poor, the prognostic factors would be helpful for the future study.
